# Structure and Principal Components Analyses Reveal an Intervarietal Fusion in Malaysian Mistletoe Fig (*Ficus deltoidea* Jack) Populations

**DOI:** 10.3390/ijms160714369

**Published:** 2015-06-24

**Authors:** Birifdzi Zimisuhara, Alireza Valdiani, Noor Azmi Shaharuddin, Faridah Qamaruzzaman, Mahmood Maziah

**Affiliations:** 1 Institute of Bioscience, Universiti Putra Malaysia, UPM Serdang, Selangor 43400, Malaysia; E-Mails: zimisuhara@gmail.com (B.Z.); fqz@upm.edu.my (F.Q.); 2 Department of Biochemistry, Faculty of Biotechnology and Molecular Sciences, Universiti Putra Malaysia, UPM Serdang, Selangor 43400, Malaysia; E-Mails: alireza.valdiani@gmail.com (A.V.); noorazmi@upm.edu.my (N.A.S.); 3 Department of Biology, Faculty of Science, Universiti Putra Malaysia, UPM Serdang, Selangor 43400, Malaysia; 4 Institute of Tropical Agriculture, Universiti Putra Malaysia, UPM Serdang, Selangor 43400, Malaysia

**Keywords:** genetic structure, genetic variation, ISSRs, STRUCTURE and cluster analyses, Principal Components Analysis (PCA)

## Abstract

Genetic structure and biodiversity of the medicinal plant *Ficus deltoidea* have rarely been scrutinized. To fill these lacunae, five varieties, consisting of 30 *F. deltoidea* accessions were collected across the country and studied on the basis of molecular and morphological data. Molecular analysis of the accessions was performed using nine Inter Simple Sequence Repeat (ISSR) markers, seven of which were detected as polymorphic markers. ISSR-based clustering generated four clusters supporting the geographical distribution of the accessions to some extent. The Jaccard’s similarity coefficient implied the existence of low diversity (0.50–0.75) in the studied population. STRUCTURE analysis showed a low differentiation among the sampling sites, while a moderate varietal differentiation was unveiled with two main populations of *F. deltoidea*. Our observations confirmed the occurrence of gene flow among the accessions; however, the highest degree of this genetic interference was related to the three accessions of FDDJ10, FDTT16 and FDKT25. These three accessions may be the genetic intervarietal fusion points of the plant’s population. Principal Components Analysis (PCA) relying on quantitative morphological characteristics resulted in two principal components with Eigenvalue >1 which made up 89.96% of the total variation. The cluster analysis performed by the eight quantitative characteristics led to grouping the accessions into four clusters with a Euclidean distance ranged between 0.06 and 1.10. Similarly, a four-cluster dendrogram was generated using qualitative traits. The qualitative characteristics were found to be more discriminating in the cluster and PCA analyses, while ISSRs were more informative on the evolution and genetic structure of the population.

## 1. Introduction

Malaysia is one of the top 12 mega biodiversity countries in the world [1]. Over the past fifty years, Malaysia has experienced an extremely rapid socio-economic growth. As a consequence, the country has lost much of its natural resources through ecosystem destruction [2]; this devastating trend leads to a lack or shortage of bioresources, including some of the valuable medicinal plants. Thus, the assessment of biodiversity is necessary to evaluate the level of conservation threats and identify the endangered species.

*Ficus deltoidea* Jack belonging to the family Moraceae, known as “mistletoe fig” in English, is considered a medicinal, as well as an ornamental plant native to Southeast Asia [3]. The plant is an evergreen shrub that can live as an epiphyte and terrestrial plant reaching up to seven meters tall. The powdered root and leaves of *F. deltoidea* have traditionally being used to treat wounds, rheumatism, diabetes, toothache, headache, cold and sore throat for centuries [4]. On the other hand, modern studies have confirmed the anti-hyperglycemic [5], anti-diabetic [6,7], antioxidant [8,9], anti-melanogenic, anti-photoaging, anti-inflammatory and anti-nociceptive [4,10], as well as wound healing [11] activity of the herb.

Despite an appreciable accessibility to the literature on pharmaceutical properties of the plant, less is known on its genetic variation, while information on biodiversity of this precious species could simultaneously contribute to the discovery of phytochemicals in the development of new medicinal drugs and also conservation of the species. Reportedly, two subspecies, 13 varieties and four forms of the species have been recognized up to now [12], eight out of those 13 varieties can be found in Malaysian forests, including var. *bilobata*, var. *angustifolia*, var. *kunstleri*, var. *intermedia*, var. *motleyana*, var. *deltoidea*, var. *kinabaluensis* and var. *trengganuensis* [13]. Local collectors of *F. deltoidea* have identified the accessions mainly based on the leaf and fruit morphology.

*F. deltoidea* probably has the most variable leaf characteristics in the genus *Ficus.* The leaf shape ranges from lanceolate, oblanceolate, spatulate, oblong, triangular, and obovate [14]. Indeed, the plant is called with various vernacular names such as Mas Cotek and Serapat (Peninsular Malaysia), Sempit-sempit (Sabah), Agoluran (Sarawak), and Tabat barito (Indonesia) due to its morphological characters in those areas; yet, they are easily distinguished from the other species of the genus with golden spots on the upper-leaf surface and glands on the underside-leaf surface [14].

Fortunately, morphological architecture and especially diverse phyllotaxis of the available varieties provides a suitable background to collect a series of quantitative and qualitative data, and also to run a set of comprehensive phylogenetic analyses. Accordingly, morphological features have been subjected to a broad investigation in the latest researches, whereas the shape and color of leaves and fruits are described highly variable [15]. More recently, Fatihah *et al.* [13] attempted to employ these features as a marker to evaluate the morphological phylogeny of the plant’s varieties in Peninsula Malaysia.

Although the efficacy of morphological markers in the study of genetics of medicinal plants has formerly been approved [16], as they are the fastest way to identify and classify an organism, but their expression is influenced by environmental conditions [17]. On the other hand, previous researchers have not concurrently employed a combination of non-molecular and molecular markers for studying the genetic variation of *F. deltoidea* varieties. Subsequently, RAPDs have showed a high genetic variation for *F. deltoidea* that should be verified by the sequence-based markers such as ISSRs [18]. Moreover, the genetic structure of the plant’s population is still a matter of ambiguity while it is a fundamental issue in population biology addressing a deeper insight into the evolutionary processes and gene flow [19]. Seemingly, a combination of morphological and DNA-based markers could be a reliable model to reveal the genetic variation and evolutionary pathway of plants [20].

The present study is the first attempt to employ molecular and morphological markers together with anb assessment of the genetic diversity and population structure of the *F. deltoidea* varieties by assembling a germplasm collection representing almost the entire Peninsular Malaysia.

## 2. Results and Discussion

### 2.1. ANOVA and Mean Comparison of the Morphological Characteristics

The ANOVA results revealed that significant differences (*p* ≤ 0.01) existed among the 30 accessions of *F. deltoidea* in terms of the eight quantitative morphological characteristics, including leaf length (LL), leaf width (LW), leaf area (LA), petiole length (PL), gold spots (GS), fruit length (FL), fruit diameter (FD) and fruit stalk (FS) as shown under “between groups” differences in Table 1.

**Table 1:** Analysis of variance of the eight quantitative morphological characteristics of the 30 *F. deltoidea* accessions.

Source of Variation	DF	Mean of Squares							
		LL	LW	LA	PL	GS	FL	FD	FS
Between groups	29	27.88 **	38.45 **	4122.83 **	13.62 **	4408.66 **	2.62 **	1.5 **	1.08 **
Within groups	270	0.29	0.27	48.74	0.22	54.27	0.02	0.00	0.02

** The mean difference is significant at 1% level; df: degree of freedom; LL: leaf length (cm); LW: leaf width (cm); LA: leaf area (cm^2^); PL: petiole length (cm); GS: number of gold spots; FL: fruit length (cm); FD: fruit diameter (cm); FS: fruit stalk length (cm).

The mean comparisons of the quantitative characteristics of the 30 *F. deltoidea* accessions are summarized in Table 2.

**Figure 1:**
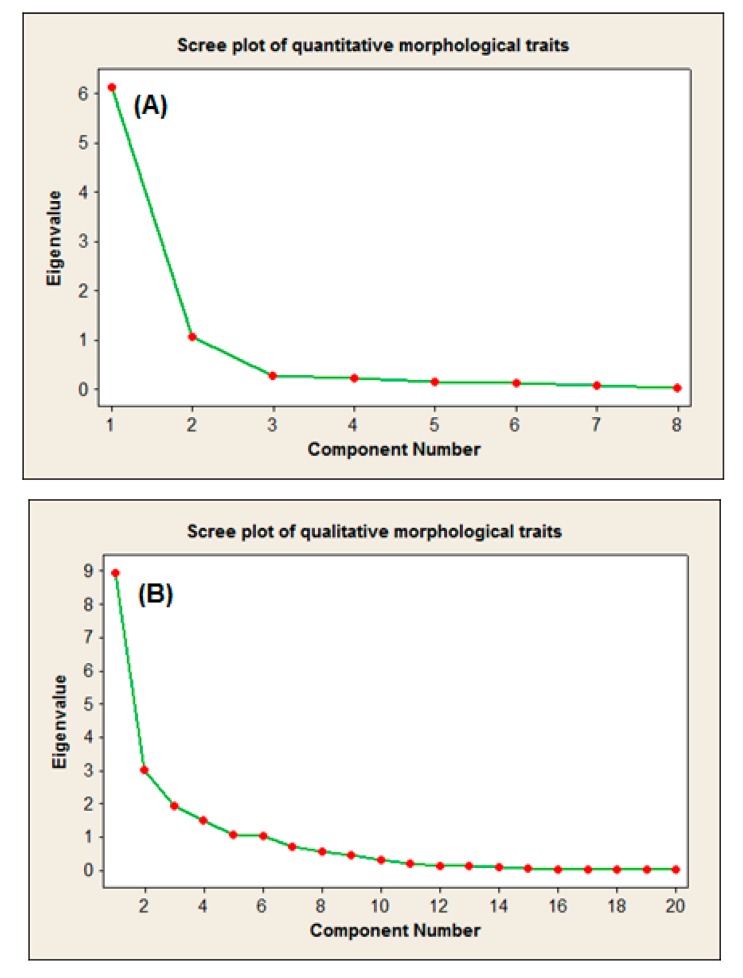
Scree plot of the eight quantitative (**A**) and 20 qualitative (**B**) morphological characteristics.

The majority of the morphological parameters showed that the collected plants have a large variation especially in the leaf characteristics. The difference between the longest leaf in accession FDKT24 (9.30 cm) and the shortest leaf in accession FDBK7 (3.41 cm) was 5.89 cm. In addition, the difference of the widest leaf in FDKT22 (8.39 cm) and the narrowest leaf in FDAK4 (1.81 cm) was 6.58 cm. The differences of LL and LW led to the significant differences in the leaf area (LA), where the difference of the broadest and smallest LA was 3.77 cm^2^ (Table 2). In fact, these results confirmed the PCA results in another way, as LA was mathematically the product of LL and LW; and consequently, LA caused the highest morphological variation (PC1 = 76.6%) among the accessions (Figure 1A). In contrast to the leaf characteristics, fruit characteristics showed little variation. The length and diameter differences of the biggest and smallest fruits were 1.65 and 1.34 cm in FDKT24 and FDBK7, respectively (Table 2).

As a comparison, the present study showed that except for leaf length and width of the var. *angustifolia* that was slightly higher in the current research, the size of leaf length, leaf width and petiole length were within the ranges of a previously conducted study by Mat *et al.* [15]. A reason of such difference could be the quality of the plant materials (herbarium collection) used in the former study while the present research studied the live specimens of the plant. Interestingly, the means of leaf length, leaf width, petiole length, and leaf area in the present study were slightly different compared to the Corner’s observations [21].

However, the measurements were more or less within the known ranges, but the impact of climate changes during the past four decades after Corner’s investigation, could be debated as one of the reasons for the observed differences in the size of the mentioned organs of the species. The ecological effects of climate changes and especially temperature on plant growth have long been regarded [22,23].

According to the results, morphological variation in the intravarietal level was slightly different from the intervarietal variation, meaning that the morphological variation among the members of the varieties was comparatively smaller (non-significant) at the intravarietal level. For instance, a non-significant difference was found for the trait LW within the members of the variety *deltoidea*. Similarly, another non-significant difference was found for the FS trait within the variety *angustifolia* and the traits GS and FW within the *deltoidea* variety. Variations among the accessions of *kunstleri* and *trengganuensis* varieties were higher than the other three varieties, whereas a significant difference at 1% level was found for all quantitative characteristics in these two varieties. The accessions of the variety *kunstleri* were the most variable members and possessed the biggest sizes in seven out of the eight quantitative traits studied.

The mean comparison results showed that the pattern of variation in the accessions collected from Pahang and Johor was mainly affected by the varietal differences rather than their geographical origin. Such a trend led to the observation of low differences (*p* ≤ 0.05) among the accessions of these two states because the accessions belonged to the same variety (var. *deltoidea)* as shown in Table 2.

**Table 2:** Mean comparison of the quantitative morphological characteristics of the 30 *F. deltoidea* accessions (Mean values ± S.E).

Accessions	LL	LW	LA	PL	GS	FL	FD	FS
**ANG**	**4.89 ± 0.72 ^j−o^**	**2.03 ± 0.22 ^m^**	**7.57 ± 1.35 ^mn^**	**0.54 ± 0.17 ^jk^**	**12.72 ± 3.34 ^j−n^**	**0.64 ± 0.08 ^l−p^**	**0.48 ± 0.07 ^k−o^**	**0.77 ± 0.14 ^e−h^**
FDAT1	4.34 ± 0.32 ^n^	2.06 ± 0.20 ^m^	7.59 ± 1.01 ^mn^	0.60 ± 0.15 ^jk^	11.50 ± 2.46 ^l−n^	0.69 ± 0.05 ^lm^	0.53 ± 0.10 ^k−m^	0.76 ± 0.08 ^e−h^
FDAT2	5.77 ± 0.44 ^jk^	2.02 ± 0.21 ^m^	8.77 ± 1.42 ^mn^	0.68 ± 0.17 ^jk^	14.60 ± 1.26 ^j−n^	0.66 ± 0.04 ^l−n^	0.43 ± 0.04 ^no^	0.88 ± 0.06 ^ef^
FDAT3	5.26 ± 0.45 ^k–m^	1.93 ± 0.15 ^m^	7.74 ± 1.16 ^mn^	0.35 ± 0.07 ^l^	8.80 ± 2.15 ^n^	0.55 ± 0.06 ^n−p^	0.45 ± 0.06 ^m−o^	0.64 ± 0.05 ^h−j^
FDAK4	4.29 ± 0.23 ^n^	1.81 ± 0.20 ^m^	7.53 ± 1.23 ^mn^	0.44 ± 0.07 ^jk^	13.30 ± 2.98 ^k−n^	0.62 ± 0.06 ^l−o^	0.57 ± 0.03 ^kl^	0.75 ± 0.10 ^e−h^
FDAT5	4.19 ± 0.19 ^no^	2.27 ± 0.08 ^m^	5.74 ± 0.50 ^mn^	0.47 ± 0.11 ^jk^	11.90 ± 2.51 ^l−n^	0.58 ± 0.10 ^m−o^	0.41 ± 0.02 ^o^	0.78 ± 0.14 ^e−h^
FDAT6	5.48 ± 0.35 ^kl^	2.06 ± 0.17 ^m^	8.06 ± 0.36 ^n^	0.66 ± 0.16 ^jk^	16.20 ± 2.90 ^j−n^	0.71 ± 0.05 ^lm^	0.50 ± 0.03 ^mn^	0.82 ± 0.24 ^e−g^
**BIL**	**3.41 ± 0.21 ^p^**	**2.10 ± 0.21 ^m^**	**3.77 ± 0.63 ^mn^**	**0.36 ± 0.07 ^k^**	**13.50 ± 2.27 ^k−n^**	**0.45 ± 0.06 ^p^**	**0.32 ± 0.04 ^p^**	**0.42 ± 0.13 ^kl^**
FDBK7	3.41 ± 0.21 ^p^	2.10 ± 0.21 ^m^	3.77 ± 0.63 ^mn^	0.36 ± 0.07 ^k^	13.50 ± 2.27 ^k−n^	0.45 ± 0.06 ^p^	0.32 ± 0.04 ^p^	0.42 ± 0.13 ^kl^
**DEL**	**4.08 ± 0.68 ^m−p^**	**3.29 ± 0.32 ^kl^**	**8.12 ± 1.78 ^mn^**	**0.72 ± 0.20 ^i−k^**	**10.97 ± 2.86 ^k−n^**	**0.65 ± 0.10 ^l−p^**	**0.61 ± 0.10 ^j−l^**	**0.50 ± 0.12 ^i−l^**
FDDP8	3.75 ± 0.30 ^op^	3.27 ± 0.33 ^l^	7.09 ± 1.31 ^n^	0.57 ± 0.12 ^jk^	10.50 ± 3.06 ^mn^	0.71 ± 0.07 ^lm^	0.56 ± 0.05 ^kl^	0.59 ± 0.08 ^ij^
FDDJ9	4.84 ± 0.52 ^m^	3.27 ± 0.28 ^l^	9.78 ± 0.87 ^mn^	0.91 ± 0.18 ^ij^	9.40 ± 2.01 ^mn^	0.72 ± 0.03 ^l^	0.68 ± 0.10 ^j^	0.53 ± 0.07 ^jk^
FDDJ10	3.65 ± 0.36 ^p^	3.34 ± 0.37 ^kl^	7.49 ± 1.75 ^mn^	0.69 ± 0.12 ^jk^	13.00 ± 2.31 ^k−n^	0.52 ± 0.03 ^op^	0.59 ± 0.08 ^k^	0.37 ± 0.07 ^l^
**TRE**	**6.33 ± 0.86 ^b−l^**	**3.86 ± 0.55 ^i−l^**	**22.13 ± 6.38 ^j−m^**	**2.20 ± 0.22 ^b−h^**	**22.97 ± 7.89 ^b−m^**	**1.40 ± 0.84 ^e−k^**	**0.97 ± 0.10 ^d−i^**	**1.20 ± 0.34 ^a−g^**
FDTT11	5.40 ± 0.50 ^kl^	4.06 ± 0.53 ^ij^	21.07 ± 4.53 ^k^	1.39 ± 0.47 ^h^	21.60 ± 7.26 ^h−j^	1.62 ± 0.20 ^ef^	0.98 ± 0.10 ^f−h^	1.43 ± 0.20 ^b^
FDTT12	5.52 ± 0.36 ^kl^	3.72 ± 0.24 ^j−l^	19.63 ± 3.67 ^kl^	2.49 ± 0.47 ^ef^	25.10 ± 7.16 ^g−i^	1.60 ± 0.10 ^ef^	0.94 ± 0.07 ^g−i^	1.21 ± 0.21 ^c^
FDTT13	6.61 ± 0.45 ^g–i^	3.62 ± 0.45 ^j−l^	22.61 ± 4.46 ^jk^	2.03 ± 0.62 ^g^	18.50 ± 5.38 ^i−l^	1.35 ± 0.29 ^ij^	1.05 ± 0.08 ^ef^	0.84 ± 0.09 ^ef^
FDTT14	6.96 ± 0.46 ^f–h^	4.03 ± 0.42 ^ij^	26.01 ± 4.99 ^h−k^	1.88 ± 0.45 ^g^	24.40 ± 3.75 ^g−i^	1.25 ± 0.09 ^jk^	0.94 ± 0.12 ^g−i^	1.19 ± 0.15 ^cd^
FDTT15	7.20 ± 0.41 ^ef^	3.84 ± 0.25 ^jk^	28.21 ± 3.97 ^h–j^	3.08 ± 0.33 ^b–d^	34.50 ± 6.65 ^ef^	1.40 ± 0.10 ^hi^	0.87 ± 0.03 ^i^	1.18 ± 0.06 ^cd^
FDTT16	6.89 ± 0.43 ^f–h^	4.51 ± 0.45 ^i^	23.71 ± 7.92 ^i−k^	3.10 ± 0.38 ^b−d^	20.10 ± 6.74 ^h−k^	1.41 ± 0.09 ^g−i^	0.94 ± 0.10 ^g−i^	1.72 ± 0.33 ^a^
FDTK17	5.75 ± 0.82 ^bjk^	3.23 ± 0.48 ^l^	13.63 ± 2.40 ^lm^	1.44 ± 0.84 ^h^	16.60 ± 2.76 ^j−m^	1.16 ± 0.10 ^k^	1.09 ± 0.05 ^de^	0.82 ± 0.08 ^e−g^
**KUN**	**7.56 ± 1.35 ^a−m^**	**6.41 ± 1.21 ^a−h^**	**48.36 ± 15.44 ^a−i^**	**2.67 ±1.05 ^a−i^**	**49.88 ± 19.93 ^a−h^**	**1.71 ± 0.25 ^a−k^**	**1.29 ± 0.26 ^a−i^**	**0.97 ± 0.31 ^a−i^**
FDKT18	5.08 ± 0.39 ^lm^	5.51 ± 0.42 ^fg^	30.37 ± 2.62 ^g−i^	1.21 ± 0.33 ^hi^	26.80 ± 7.00 ^gh^	1.53 ± 0.09 ^fg^	0.91 ± 0.05 ^hi^	1.06 ± 0.15 ^d^
FDKT19	6.52 ± 0.54 ^hi^	5.55 ± 0.57 ^fg^	35.96 ± 7.25 ^fg^	3.48 ± 0.64 ^b^	31.50 ± 5.36 ^fg^	1.28 ± 0.14 ^jk^	1.29 ± 0.09 ^c^	1.72 ± 0.10 ^a^
FDKT20	6.12 ± 0.68 ^ij^	5.02 ± 0.80 ^h^	32.69 ± 4.50 ^f−h^	2.01 ± 0.33 ^g^	30.90 ± 11.27 ^fg^	1.68 ± 0.13 ^de^	1.00 ± 0.09 ^fg^	0.90 ± 0.17 ^e^
FDKT21	7.10 ± 0.56 ^e–g^	6.01 ± 0.77 ^ef^	44.23 ± 9.52 ^c−e^	2.92 ± 0.64 ^c−e^	63.30 ± 8.96 ^b^	1.81 ± 0.17 ^bc^	1.28 ± 0.10 ^c^	1.28±0.22 ^c^
FDKT22	8.38 ± 0.64 ^bc^	8.40 ± 0.50 ^a^	68.04 ± 10.18 ^a^	3.26 ± 0.58 ^bc^	61.80 ± 11.92 ^b^	1.80 ± 0.10 ^b−d^	1.63 ± 0.14 ^a^	0.90 ± 0.08 ^e^
FDKK23	6.59 ± 0.76 ^g–i^	5.32 ± 0.54 ^gh^	39.07 ± 7.85 ^ef^	1.24 ± 0.26 ^hi^	44.00 ± 6.72 ^cd^	1.70 ± 0.40 ^c−e^	0.98 ± 0.16 ^f−h^	0.89 ± 0.15 ^e^
FDKT24	9.31 ± 0.56 ^a^	5.98 ± 0.45 ^ef^	65.01 ± 8.29 ^ab^	2.65 ± 0.87 ^de^	63.70 ± 6.78 ^b^	2.10 ± 0.16 ^a^	1.67 ± 0.14 ^a^	0.81 ± 0.08 ^e−g^
FDKT25	8.57 ± 0.73 ^b^	5.85 ± 0.59 ^f^	42.83 ± 14.07 ^de^	4.66 ± 0.55 ^a^	45.10 ± 4.25 ^cd^	1.70 ± 0.10 ^c−e^	1.52 ± 0.10 ^f−h^	1.21 ± 0.12 ^c^
FDKT26	8.02 ± 0.54 ^cd^	6.75 ± 0.66 ^cd^	59.50 ± 13.49 ^b^	3.08 ± 0.56 ^b−d^	92.70 ± 20.15 ^a^	1.61 ± 0.11 ^ef^	1.45 ± 0.11 ^b^	0.68 ± 0.23 ^g−i^
FDKT27	8.71 ± 1.06 ^b^	6.45 ± 1.34 ^de^	50.79 ± 15.98 ^c^	2.98 ± 0.64 ^cd^	46.50 ± 12.96 ^c^	1.50 ± 0.02 ^f−h^	1.12 ± 0.04 ^de^	0.73 ± 0.12 ^f−h^
FDKT28	8.82 ± 0.77 ^b^	7.08 ± 0.76 ^c^	48.83 ± 6.63 ^cd^	2.49 ± 0.49 ^ef^	38.70 ± 9.04 ^de^	1.92 ± 0.17 ^b^	1.26 ± 0.09 ^c^	0.75 ± 0.14 ^e−h^
FDKK29	7.59 ± 0.81 ^de^	7.61 ± 1.02 ^b^	46.84 ± 7.63 ^cd^	2.08 ± 0.61 ^fg^	43.00 ± 2.75 ^cd^	1.89 ± 0.03 ^b^	1.47 ± 0.04 ^b^	0.81 ± 0.08 ^e−g^
FDKK30	7.54 ± 0.44 ^de^	7.61 ± 0.32 ^b^	64.51 ± 12.05 ^ab^	2.68 ± 0.72 ^de^	60.50 ± 11.16 ^b^	1.69 ± 0.09 ^c−e^	1.16 ± 0.09 ^a^	0.85 ± 0.12 ^ef^

ANG = var. *Angustifolia*; BIL = var. *Bilobata*; DEL = var. *Deltoidea*; TRE = var. *Trengganuensis*; KUN = var. *Kunstleri*; LL: leaf length (cm); LW: leaf width (cm); LA, leaf area (cm^2^); PL: petiole length (cm); GS: number of gold spots; FL: fruit length (cm); FD: fruit diameter (cm); FS: fruit stalk length (cm); The bold rows represent the mean of each variety. Similar letters indicate no significant difference (*p* ≤ 0.05).

### 2.2. Principal Components Analyses (PCA) Using Morphological Data

#### 2.2.1. Scree Plot-Based Representation of the PCA

Even though the ANOVA results confirmed the suitability of these traits to expose the morphological differences of the accessions, a further step was taken to illustrate the effectiveness of each characteristic in the population’s variation [24]. Principal components analysis is a powerful approach in germplasm collections that allows a better understanding on the structure of the entire collection. PCA makes it possible to identify the most suitable variables among the studied accessions [25,26,27]. Therefore, the most important outcome of the scree plot-based PCA was to ease detecting the principal components with an Eigenvalue greater than an arbitrary value *K* = 1. The illustrative feature of the scree plot-based PCA was mostly due to the simple and one-dimensional nature of the generated graphs. These principal components (PCs) can be utilized to calculate the morphological distances of the accessions in the future studies [28,29].

The component matrices of the quantitative-based PCA revealed two main principal components. In PC1, LA and FD with Eigenvalues of 6.13 and 1.06 caused 76.7 and 13.1% of the total variation in the quantitative data, respectively. After these two characteristics, FS was the most effective trait of the PC2 (Figure 1A). The PCA of the qualitative characteristics resulted in generating six PCs in which shape of leaf apex (LAS), tree habit (THA), visible glands on the upper-leaf surface (LGV), fruit scale color (FSC), leaf venation of the upper-leaf surface (LVU), and gland color (LGC) with the Eigenvalues of 8.94, 3.02, 1.91, 1.51, 1.06 and 1.02, respectively served as the most effective PCs. These six PCs caused a total of 87.4% of qualitative variation in the morphology of the 30 accessions (Figure 1B). These results indicate that LA, FD, LGA, THA and LGV could be suitable candidates to investigate the morphological variation of the species in future studies.

#### 2.2.2. Biplot-Based Representation of the PCA

As mentioned in the Section 2.2.1, the scree plots based on the morphological characteristics revealed the exact proportion of each quantitative and qualitative trait (component) in the total variation without any further information. Unlike the one-dimensional visualization of scree plot, biplot is a two-dimensional approach for grouping accessions considering the associated characteristics.

**Figure 2:**
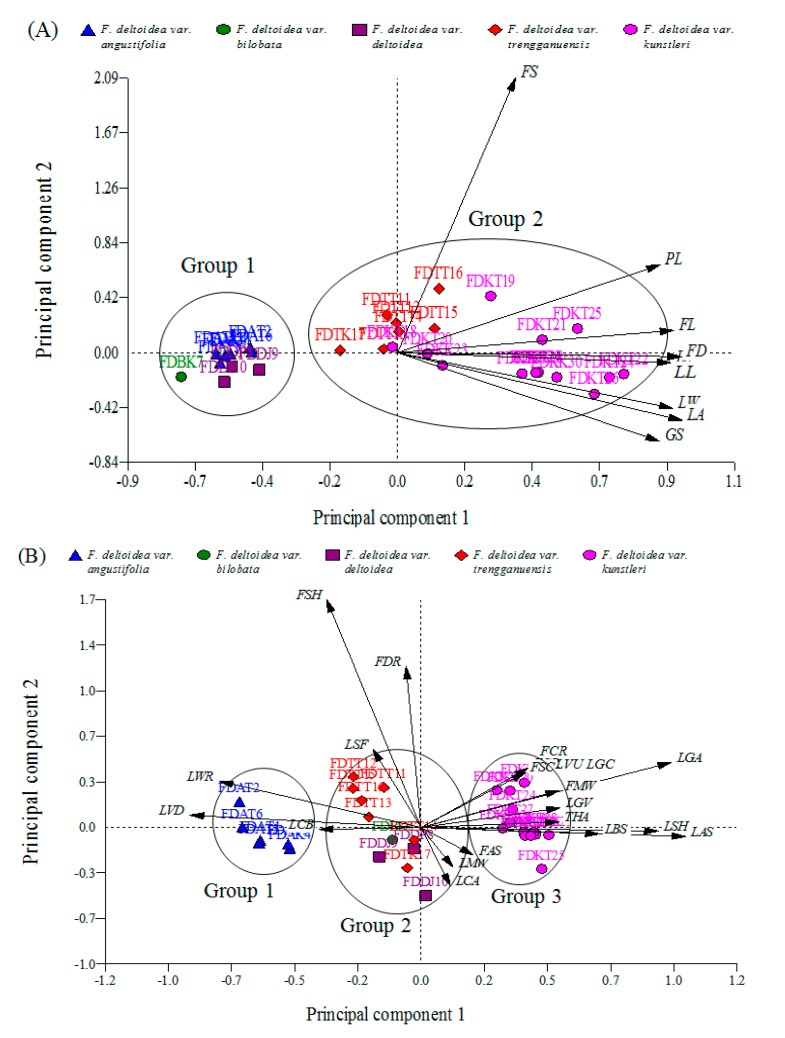
Biplot of the 8 quantitative (**A**) and 20 qualitative (**B**) morphological characteristics. PC: Principal component.

As the most compelling result, the Euclidean biplot showed that the distribution of the accessions in quantitative-based PCA was focused on two groups (Figure 2A). Group 1 included the accessions of *F. deltoidea* var. *angustifolia*, var. *deltoidea* and var. *bilobata*. They were closely grouped on the negative end of the PC1. Group 2 included the accessions of *F. deltoidea* var. *trengganuensis* and var. *kunstleri*. The accessions of the second group were widely distributed on the negative and positive sides of the PC1 and PC2. All the eight vectors of the quantitative characteristics were located on the positive side of the PC1 (Figure 2A). Proximity of the accessions to the vectors was in agreement with strong influence of those characteristics (vectors) on the closest accessions. For example, accession FDTT16 closed to the FS vector got the longest fruit stalk. Similarly, most of the group 2 accessions belonging to var. *kunstleri* and var. *trengganuensis* with bigger leaves and fruits were situated in a closer position to the leaf- and fruit-related vectors (Figure 2A). In contrast, group 1 accessions, which were located opposite to the direction of these vectors, were smaller in leaf and fruit size. Both of the biplots showed no particular pattern of grouping according to the geographical origins of the accessions, but the accessions were divided into different groups based on their varietal sources (Figure 2A,B).

Remarkably, a similar trend was evident in the PCA-based studies on *Ficus carica* as a close relative to *F. deltoidea*, whereas no correlation existed between the *F. carica* varieties and their geographical origin [30,31,32]. Furthermore, the quantitative-based biplot divided the accessions into two groups by separating the var. *deltoidea*, var. *bilobata* and var. *angustifolia* as group 1 from those of var. *kunstleri* and var. *trengganuensis* as group 2 (Figure 2A).

Conversely, the qualitative-based biplot led to constructing three distinct groups by segregating the varieties *angustifolia* and *kunstleri* as group 1 and 3, respectively, and, mixing the other three varieties together into group 2 (Figure 2B). Beside the presentation of a new pattern of grouping, the qualitative-based biplot revealed the first sign of the existence of an intervariatal genetic passage among the studied population of *F. deltoidea*. To date, principal components analysis using morphological and molecular evidence has successfully been employed to prove the hybridization and introgression in different plant and animal species [33,34].

### 2.3. Morphological and Molecular Variation of the F. deltoidea Varieties Based on the Cluster Analysis

#### 2.3.1. Cluster Analysis Based on the Quantitative Morphological Markers

**Figure 3:**
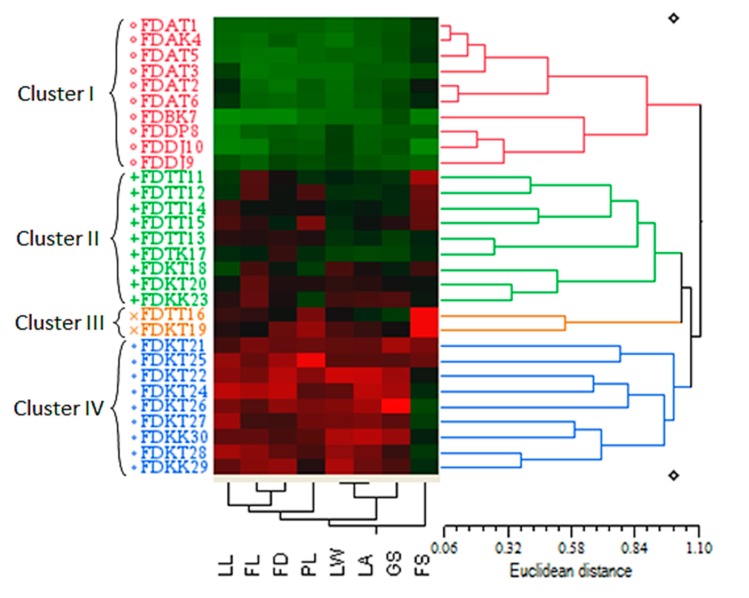
Quantitative-based dendrogram of the 30 *Ficus deltoidea* accessions generated by the UPGMA clustering method. The genetic similarity matrices are shown by Euclidean distance.

The cluster analysis of the 30 accessions based on the UPGMA method and using the eight quantitative characteristics led to grouping the accessions into four main clusters with a Euclidean distance ranged between 0.06 and 1.10 (Figure 3). Cluster I (red cluster) included the ten accessions belonging to the varieties *angustifolia*, *bilobata* and *deltoidea* regardless of their geographical origin. Cluster II (green cluster), was composed of six accessions of var. *trengganuensis* and three accessions of var. *kunstleri*. The blue cluster (cluster IV) was unexceptionally comprised of the var. *kunstleri* accessions. The orange cluster (cluster III), as the most low-populated cluster, contained two accessions of two different varieties viz. *trengganuensis* and *kunstleri* (FDTT16 and FDKT19) both from Terengganu. Therefore, unlike the other three clusters, the orange cluster (cluster III) responded significantly to geographical distribution. In addition, geographical integration occurred in subclusters’ levels as well. For example, the accessions FDKT28 (from Terengganu) and FDKK29 (from Kelantan) both belonging to var. *kunstleri*, were mixed in the same subcluster of the blue cluster (cluster IV). The same condition was evident in one of the green subclusters (in cluster II), whereas the accessions FDKT20 (from Terengganu) and FDKK23 (from Kelantan) both belonging to the var. *kunstleri* were placed in the same subcluster (Figure 3). The results of the cluster analysis based on the quantitative characteristics were in agreement with the PCA analysis of the population using the same characteristics, whereas the members of the red cluster (cluster I) in Figure 3 (consisting of the *angustifolia*, *bilobata* and *deltoidea* varieties) were present at the group 1 in Figure 2A.

Furthermore, the cluster analysis was found more specific in discriminating the intermediate accessions considering its morphometric-based color map. Phenotypically, it was postulated that the accessions of the green and orange clusters (clusters II and III) can be the intermediate forms of the red and blue clusters’ accessions (cluster IV) through sharing the black, green and red squares of the color map together (Figure 3). To be more explicit, the accessions of the clusters II and III possessed a combination of the morphological traits of the red and blue (clusters I and IV) clusters’ members. In another word, these accessions probably could be considered as the crossbreeds of the red and blue (clusters I and IV) clusters’ varieties. Nevertheless, the accession FDTT16 belonging to the var. *trengganuensis* collected from Terengganu was the best candidate for detecting a genetic confluence point of the population. Such observation complies with the possibility of the intraspecific hybridization in this plant. The latter interpretation of the cluster analysis is in line with the recent employment of the technique to prove the hybridization and introgression phenomena in modern biology [35,36,37,38].

#### 2.3.2. Cluster Analysis Based on the Qualitative Morphological Markers

**Figure 4:**
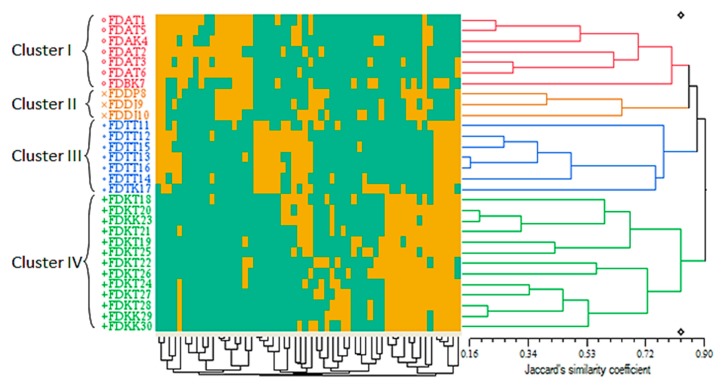
Qualitative-based dendrogram of the 30 *Ficus deltoidea* accessions generated by the UPGMA clustering method. The green and yellow squares of the color map refer to the binary representation of the two possibilities for each qualitative characteristic in every accession (0 and 1 respectively). The genetic similarity matrices are shown by Jaccard’s similarity coefficient.

Similar to the quantitative-based cluster analysis, a four-cluster dendrogram was generated using the 20 qualitative traits on the basis of Unweighted Pair Group Method with Arithmetic Mean (UPGMA) method. The Jaccard’s similarity coefficients [39] were ranged between 0.16 and 0.90 (Figure 4).

The number of the clusters was in agreement with the quantitative-based dendrogram. Once again, the arrangement of the accessions in the generated clusters corresponded to their varietal source rather than their geographical distribution. Surprisingly, the qualitative characteristics were more efficient in differentiating the three accessions of the var. *deltoide*a by constructing the orange cluster (cluster II) (Figure 4). However, the quantitative characteristics were unable to separate this variety, so that the three accessions of this variety were combined with the seven accessions of the *angustifolia* and *bilobata* varieties in the red cluster (cluster I) (Figure 3). In addition, the qualitative leaf characteristics (such as leaf shape) played a more differentiative role than the quantitative leaf characteristics (such as leaf size) in separating var. *deltoidea* from var. *angustifolia* and var. *bilobata*. The orange cluster (cluster II) corresponded to the geographical origin of the accessions to some extent, in which the three accessions, including FDDP8 (from Pahang), as well as FDDJ9 and FDDJ10 (from Johor) were placed in this cluster (cluster II) together. An explanation of this event is that the accession FDDP8 was collected from a very southern part of Pahang state neighboring Johor, where the other two accessions of FDDJ9 and FDDJ10 were collected. Despite a minor geographical distance, the mentioned three accessions formed the orange cluster (cluster II) because of their common varietal source (var. *deltoidea*).

Although the color map of the qualitative-based dendrogram was comprised of two colors due to the binary conversion of the qualitative data (as mentioned in the Experimental Section 3.3.2), the proportion of the yellow and green squares provides a clue about the importance of each qualitative trait in the formation of the clusters and their subclusters, as well as on the arrangement of the accessions in each cluster. Accordingly, the members of the blue cluster (cluster III) could be considered as the intermediate forms of the green, red and orange clusters (clusters I and II) (Figure 4). Hence, from the evolutionary point of view, the latter outcome is highly in accordance with the quantitative-based dendrogram, as shown in Figure 3.

The current pattern of the qualitative-based cluster analysis was largely matched with the previous results explained by Fatihah *et al.* [13], except that in the present study var. *deltoidea* was closer to var. *angustifolia* compared to the former study, in which var. *deltoidea* was closer to var. *bilobata*. As a final point, the qualitative-based dendrogram suggests that the clustering of diverse *F. deltoidea* genetic materials is not related to their geographical origin but it refers to specific phenotypic characters (Figure 4). A similar template has been shown in sweet potato, where the cluster analysis of its cultivars was not associated with their specific agroecological zones [40]. Such a situation is expected since the transfer of genetic materials and propagation of the same resources has taken place in some areas.

#### 2.3.3. Cluster Analysis Based on the ISSR Markers

**Figure 5:**
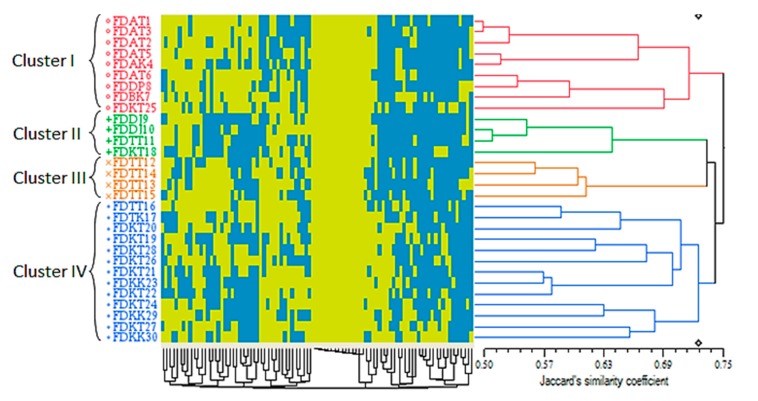
ISSR-based dendrogram of the 30 *Ficus deltoidea* accessions generated by the UPGMA clustering method. The blue and yellow squares of the color map indicate the absence (0) and presence (1) of the ISSR loci in each accession, respectively. The genetic similarity matrices are shown by Jaccard’s similarity coefficient.

The ISSR-based cluster analysis of the 30 accessions resulted in different outcomes compared to the morphological-based cluster analyses when a dendrogram consisted of four main clusters with a Jaccard’s similarity coefficient ranged between 0.50 and 0.75 was generated (Figure 5). While the number of clusters resembled the other two dendrograms with a different pattern, the range of the similarity coefficients was decreased in the molecular-based cluster analysis.

A subsidiary output of the qualitative- and ISSR-based clustering analyses was the performance of the Jaccard’s similarity coefficient in the arrangement of the accessions in the generated clusters. Since the present study was focused on studying the genetic relationship of the varieties of a single species, detecting the actual differences was important to avoid any pseudo-diversity. Technically, Jaccard’s, Sorensen-Dice and Nei coefficients exclude negative matches or so-called “negative co-occurrences” (the 0-0 situation in binary data). The consequence of such a feature is the detection of a more precise similarity compared to the methods lacking this characteristic, e.g., simple matching (SM) [41]. Thus, application of the Jaccard’s similarity coefficient has been preferred for studying the closely related organisms [42]. Furthermore, the ISSR markers were able to differentiate the var. *deltoidea* from var. *angustifolia* and var. *bilobata* to some extent (Figure 5), while these varieties were mixed together in the red cluster (cluster I) of the quantitative-based dendrogram (Figure 3). Regardless of the dominant feature of both ISSR and RAPD markers, Jaccard’s coefficient made by the ISSR markers (0.50–0.75) was distant of those generated by RAPD markers (0.2–1.0) in *F. deltoidea* varietis [18]. Nontheless, the only common point of these markers was generating four clusters for a similar number of accessions (30 *vs.* 26)

#### 2.3.4. Correlation of Similarity Matrices between Morphology and ISSR Marker Systems

The Mantel test correlation [43] showed a positive correlation between morphology and ISSR marker systems. The correlation coefficient of the ISSRs and quantitative morphological traits was (*r* = 0.49 **), while the correlation of the ISSRs and qualitative morphological traits was (*r* = 0.39 **). The (*r*) values indicated a moderate correlation between the morphological characteristics and the amplified ISSRs in this study.

### 2.4. ISSR-PCR and Population Structure

Of nine ISSR primers used to screen the DNA pattern of the 30 accessions, seven primers produced polymorphic ISSR-PCR products, while two of them (ISSR20 and ISSR30) were found to be monomorphic (Table 3).

The primers used resulted in generating a total of 106 ISSR loci (bands) of which 74 were found polymorphic. The lowest and highest number of loci was generated by the primers ISSR20 (6 loci) and UBC823 (15 loci), respectively. The sizes of the generated loci were ranged between 155 and 2544 bp. The Shannon indices showed that the primer ISSR25 (0.559 ± 0.202) and ISSR16 (0.361 ± 0.288), caused the highest and lowest percentages of polymorphism in the studied accessions (Table 3).

**Figure 6:**
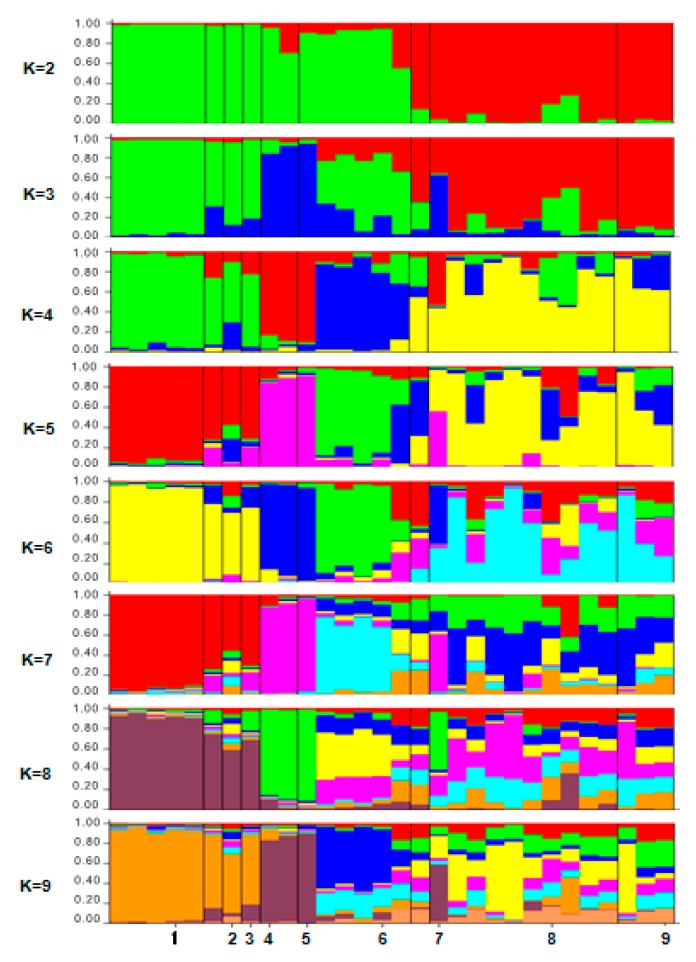
The population memberships of the inspected species groups for *a priori* defined number of clusters *K* = 1–9 inferred by the STRUCTURE software (Pritchard Lab, CA, USA). Each accession is represented by a vertical column divided into K colored segments that represent the individual’s estimated membership fractions in K clusters. Black lines separate the nine populations. The numbers at the bottom of the graph refer to the population codes mentioned as in Table 1.

The structure analysis of *Ficus deltoidea* was initially performed using the maximum number of population (*K* = 9) as shown in Figure 6.

**Table 3:** Polymorphic content of 10 ISSR primers amplified on *F. deltoidea*.

Primer	Sequence	Tm (°C)	Size Range (bp)	NAL *	NPL **	Shannon Index	Polymorphism (%)
ISSR16	(CAC)_3_GC	38.0	174–1551	14	10	0.361 ± 0.288	71.4
I841	(GA)_8_CC	50.3	420–1926	14	12	0.445 ± 0.243	85.7
ISSR25	(AC)_8_GA	48.0	155–1524	11	10	0.559 ± 0.202	90.9
UBC815	(CT)_8_G	47.0	218–1155	11	9	0.411 ± 0.254	81.8
UBC816	(CA)_8_T	50.0	158–1341	14	11	0.390 ± 0.277	78.5
UBC823	(TC)_8_C	48.0	173–1239	15	13	0.473 ± 0.233	86.7
UBC806	(TA)_8_G	27.8	166–1564	10	9	0.504 ± 0.212	90.0
ISSR20	(AC)_7_TA	45.8	700–1792	6	0	–	0
ISSR30	(AC)_8_	43.4	385–2544	11	0	–	0
10	–	–	155–2544	106/15.14	74/8.2	0.449 ± 0.247	65.0

* Number of amplified loci; ** Number of polymorphic loci.

**Figure 7:**
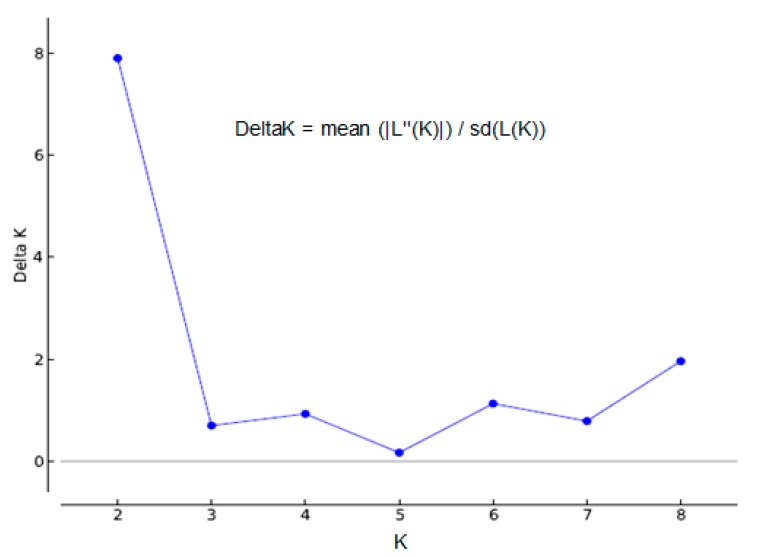
The detection of the true number of clusters (the most likely value of K) inferred by the STRUCTURE software and set ΔK = mean (|L″(K)|)/sd (L(K)) as a function of *K*. ΔK achieves its highest peak when K = 2, generated by the STRUCTURE HARVESTER, and based on the approach of Evanno *et al.* [45].

Nevertheless, the most likely numbers of population was estimated according to the highest peak of ΔK value (K = 2) inferred from the performed structure analysis (Figure 7). The ΔK value is an *ad hoc* quantity related to the second order rate of change of the log probability of data with respect to the number of clusters [44].

**Figure 8:**
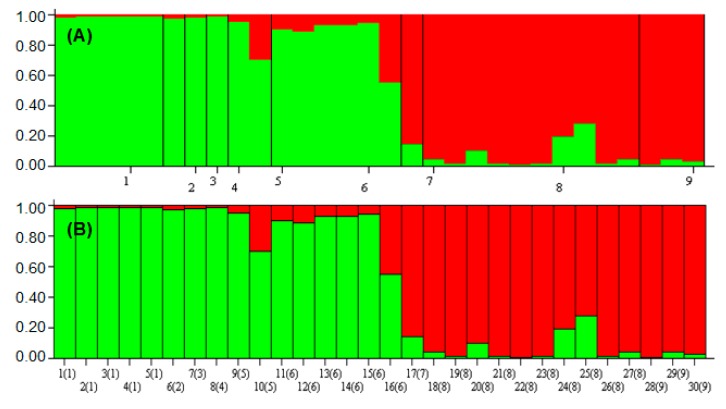
Population- (**A**) and accession-based (**B**) STRUCTURE graph. Populations and accessions are labeled below each figure. The numbers on the *x*-axis of graph (**A**), as well as the numbers inside the parentheses of the *x*-axis of graph (**B**) refer to the population codes mentioned as in Table 1. The numbers besides the parentheses in the graph (**B**) refer to the number of accessions.

The structure analysis led to the emergence of two distinct populations of *F. deltoidea*, shown with green and red colors (Figure 8A), and the occurrence of gene flow among the accessions was detected to some extent. The highest degree of this genetic interference was related to the FDDJ10, FDTT16 and FDKT25 accessions shown as 10(5), 16(6) and 25(8) in Figure 8B.

These three accessions are possibly the intervarietal fusion points of the plant’s population, genetically. The allogamous mating system of the plant could be an explanation for observing such a genetic structure especially about the FDTT16 and FDKT25 accessions due to their geographical proximity in Terengganu (Table 1). Despite this, the genetic composition of the accessions such as FDDJ10 from Johor can be judged more precisely by using codominant markers such as simple sequence repeats (SSRs), and the PCR-based restriction fragment length polymorphism (PCR-RPLPs). The interpretation of the structure analysis of the present exploration is in a close relation with those conclusions conducted by Egger *et al.* [46], whereas introgressive hybridization has been tracked by exploiting a combination of PCA, cluster and structure analyses.

### 2.5. Evolutionary Hypothesis and Mating System of F. deltoidea

Most of the species of the *Ficus* genus are known as allogamous plants and their pollination is critically dependent on the host-specific fig pollinator wasps from the family Agaonidae [47,48]. The absence of these pollinators has been emphasized as an obstacle in spreading the *F. deltoidea* species in the areas such as Hawaii [49].

As mentioned, to illustrate the accurate rate of heterozygosity in the accessions such as FDDJ10, FDTT16 and FDKT25 they should be subjected to codominant markers. However, regarding the current results, it can be roughly predicted that the reason of genetic interaction in the accession FDDJ10 can be attributed to the pollen donators of the adjacent states such as Melaka, Pahang and Negeri Sembilan. The pollination process could be facilitated with the help of fig wasps as the main species-specific pollinators of the plant. In view of an intricate mutual coevolution of fig (*Ficus*, Moraceae) and fig wasps (Agaoninae, Agaonidae, Chalcidoidea) for over approximately 90 million years, occurrence of such an event is justifiable [50]. Nonetheless, the question arises here why the same did not happen to another accession (FDDJ9) collected from Johor. Probably, the answer should be searched in the plant’s density in the Muar area where the FDDJ9 accession was collected. As an ecological point, establishment and maintenance of the short-lived and species-specific population of the wasp pollinator requires a minimum number of fig populations. The concept is referred to as the critical minimum number of trees or critical population size (CPS). Temporal gaps occur among flowering trees when plant density drops below the CPS range. These gaps are often unbridgeable for pollinators, and lead to their local extinction [51]. In this regard, Poore [52] who enumerated the upper canopy trees only, encountered with two species of *Ficus*, that each was represented by a single individual in 23 ha. The extinction of the local wasp’s population in turn decreases the rate of cross-pollination compared to self-pollination. Such a trend tends to increase the rate of homozygosity (or decrease the observed heterozygosity) in the short term. Obviously, conservation of the plant will be threatened by decreasing genetic diversity in the long term.

Apparently, the mating system of the species (allogamy) could be an explanation for detecting such genetic heterogeneity in the studied population of *F. deltoidea*. Despite the dominant feature of the ISSR markers, a comprehensive conclusion on the exact percentage of heterozygosity is almost impossible. For this reason, conducting similar experiments using co-dominant markers, such as SSR markers, is inevitably required. The higher rate of observed heterozygosity has been accentuated as a residue of intercrossing in allogamous plants [53].

## 3. Experimental Section

### 3.1. Plant Materials

**Figure 9:**
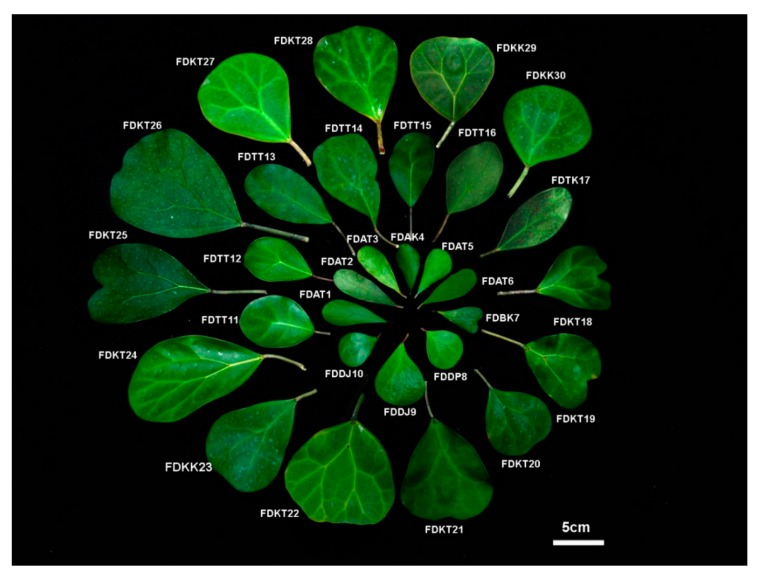
Schematic presentation of leaf morphology variation in the 30 *F. deltoidea* accessions collected from Peninsular Malaysia.

Five varieties of *Ficus deltoidea* consisting of 30 accessions were selected based on the morphological dissimilarities from different states of Peninsular Malaysia (Table 4). The accessions were identified based on the Corner’s guidelines [21]; mainly using the leaf shape and structure, as shown in Figure 9. The collected plants were maintained in a glass house at Universiti Putra Malaysia (UPM) to reduce the environmental effects on the future sampling and measurements.

### 3.2. Ethics Statement

The plant accessions used in the present study were collected from private lands and transferred into UPM as a part of the university’s germplasm collection. In addition, the field studies did not involve endangered or protected species. Hence, no specific permissions were required for these locations/activities. The GPS coordinates of the collection sites, as well as the origin of the accessions, have been presented with detail in Table 4.

**Table 4:** List of the 30 *F. deltoidea* accessions.

Population Code *	Accession	Botanical Name	Origin	Latitude	Longitude
1	FDAT1	*F. deltoidea* var. *angustifolia*	Terengganu	5° 39.086 N	102° 84.198 E
1	FDAT2	*F. deltoidea* var. *angustifolia*	Terengganu	5° 60.775 N	102° 74.873 E
1	FDAT3	*F. deltoidea* var. *angustifolia*	Terengganu	5° 39.086 N	102° 84.198 E
2	FDAK4	*F. deltoidea* var. *angustifolia*	Kelantan	5° 97.854 N	102° 42.650 E
1	FDAT5	*F. deltoidea* var. *angustifolia*	Terengganu	5° 60.775 N	102° 74.873 E
1	FDAT6	*F. deltoidea* var. *angustifolia*	Terengganu	5° 52.281 N	102° 93.927 E
3	FDBK7	*F. deltoidea* var. *bilobata*	Kelantan	5° 97.854 N	102° 42.650 E
4	FDDP8	*F. deltoidea* var. *deltoidea*	Pahang	2° 90.220 N	102° 89.700 E
5	FDDJ9	*F. deltoidea* var. *deltoidea*	Johor	1° 89.186 N	102° 76.717 E
5	FDDJ10	*F. deltoidea* var. *deltoidea*	Johor	1° 93.317 N	103° 18.551 E
6	FDTT11	*F. deltoidea* var. *trengganuensis*	Terengganu	5° 39.086 N	102° 84.198 E
6	FDTT12	*F. deltoidea* var. *trengganuensis*	Terengganu	5° 39.086 N	102° 84.198 E
6	FDTT13	*F. deltoidea* var. *trengganuensis*	Terengganu	5° 39.086 N	102° 84.198 E
6	FDTT14	*F. deltoidea* var. *trengganuensis*	Terengganu	5° 39.086 N	102° 84.198 E
6	FDTT15	*F. deltoidea* var. *trengganuensis*	Terengganu	5° 52.281 N	102° 93.927 E
6	FDTT16	*F. deltoidea* var. *trengganuensis*	Terengganu	5° 60.775 N	102° 74.873 E
7	FDTK17	*F. deltoidea* var. *trengganuensis*	Kelantan	5° 97.854 N	102° 42.650 E
8	FDKT18	*F. deltoidea* var. *kunstleri*	Terengganu	5° 39.086 N	102° 84.198 E
8	FDKT19	*F. deltoidea* var. *kunstleri*	Terengganu	5° 52.281 N	102° 93.927 E
8	FDKT20	*F. deltoidea* var. *kunstleri*	Terengganu	5° 39.086 N	102° 84.198 E
8	FDKT21	*F. deltoidea* var. *kunstleri*	Terengganu	5° 60.775 N	102° 74.873 E
8	FDKT22	*F. deltoidea* var. *kunstleri*	Terengganu	5° 39.086 N	102° 84.198 E
9	FDKK23	*F. deltoidea* var. *kunstleri*	Kelantan	5° 97.854 N	102° 42.650 E
8	FDKT24	*F. deltoidea* var. *kunstleri*	Terengganu	5° 39.086 N	102° 84.198 E
8	FDKT25	*F. deltoidea* var. *kunstleri*	Terengganu	5° 39.086 N	102° 84.198 E
8	FDKT26	*F. deltoidea* var. *kunstleri*	Terengganu	5° 39.086 N	102° 84.198 E
8	FDKT27	*F. deltoidea* var. *kunstleri*	Terengganu	5° 60.775 N	102° 74.873 E
8	FDKT28	*F. deltoidea* var. *kunstleri*	Terengganu	5° 39.086 N	102° 84.198 E
9	FDKK29	*F. deltoidea* var. *kunstleri*	Kelantan	5° 97.854 N	102° 42.650 E
9	FDKK30	*F. deltoidea* var. *kunstleri*	Kelantan	5° 97.854 N	102° 42.650 E

* The population codes are given to each accession for STRUCTURE analysis only in this study.

### 3.3. Morphological Characteristics

A total of 28 morphological characteristics, including eight quantitative and 20 qualitative traits were measured for further analyses. Of these, one qualitative character was considered for tree habit. Moreover, thirteen qualitative and five quantitative leaf characteristics along with six qualitative and three quantitative fruit characteristics were taken into consideration. The fully expanded leaves of five different nodes were measured in mature branches. Fruit characteristics were measured in fully developed ripening fruits. Data were collected according to the instructions of the International Plant Genetic Resources Institute (IPGRI) and the Centre International de Hautes Etudes Agronomiques Méditerranéennes (CIHEAM) for fig genetic resources characterization [54].

#### 3.3.1. Quantitative Data Collection

The quantitative traits consisted of leaf length (LL, cm), leaf width (LW, cm), leaf area (LA, cm^2^), petiole length (PL, cm), number of gold spots (GS), fruit length (FL, cm), fruit diameter (FD, cm), and fruit stalk length (FS, cm) were measured according to the units in the SI system. Each quantitative trait was measured from ten randomly selected leaves and fruits from each plant. Leaf length and width, petiole length, fruit length and diameter, as well as fruit stalk length, were measured using a Vernier Caliper. The leaf area was measured by following the Pandey and Singh’s method [55].

#### 3.3.2. Qualitative Data Collection

The qualitative characteristics included of the tree habit (THA), leaf shape (LSH), shape of leaf apex (LAS), shape of the leaf base (LBS), shape of the leaf surface (LSF), maximum width of the leaf (LMW), leaf length:width ratio (LWR), color of the upper-leaf surface (LCA), color of the underside-leaf surface (LCB), gland color (LGC), amount of glands (LGA), visible glands on the upper-leaf surface (LGV), leaf venation of the upper-leaf surface (LVU), vein began to diverge (LVD), fig shape (FSH), maximum width of fig (FMW), fig apex shape (FAS), fig length: diameter ratio (FDR), color of the ripening fig (FCR), and scale color (FSC). As a measurement issue, the qualitative characteristics were more complicated (subjective) to evaluate because of their nonparametric nature. These traits were coded differently as binary and non-binary for the cluster analysis and PCA, respectively. The strategy of converting the data into binary form was essential for generating a two-color map in the qualitative-based dendrogram to avoid any confusion. Otherwise, many colors were included in the color map while similar colors of different columns would not represent the same description. The use of binary data led to generating a two-color illustrative color map with 56 columns for the related dendrogram. On the other hand, adapting these traits in the binary form in PCA analysis was hampered by existence of more than two possibilities for some of the traits. As such, the characteristics were given parametric codes from 1 to 4 to make them more sensible as shown in Table 5. The mentioned strategy resulted in producing a scree plot for the quantitative characteristics with only 20 PCs as desired.

### 3.4. Molecular Characterization and Data Collection

A set of nine Inter Simple Sequence Repeat (ISSR) primers (Integrated DNA Technologies Inc., Singapore, Singapore) was employed for DNA fingerprinting of the 30 *F. deltoidea* accessions (Table 3). Scoring the ISSR loci was performed by setting the UVIDoc software version 99.02 on the manual mode for the actual band sizing. The code “1” was given for the presence of the loci and “0” for their absence.

**Table 5:** List and descriptions of the 28 qualitative and quantitative morphological characteristics.

Characters	Abbreviation	Coding Description
Tree habit	THA	1, erect; 2, leaning
Leaf shape *	LSH	1, spatulate; 2, elliptic/oval; 3, obovate
Shape of leaf apex	LAS	1, obtuse; 2, rounded; 3, truncate; 4, obcordate
Shape of the leaf base	LBS	1, attenuate; 2, narrowly cuneate; 3, cuneate
Type of the leaf surface	LSF	1, flat; 2, vein impressed; 3, margin dented
Maximum width of the leaf	LMW	1, 1/3 to 2/3 from leaf base; 2, more than 2/3 from leaf base
Leaf length: width ratio *	LWR	1, 1:1; 2, 1.5:1; 3, 2:1; 4, 3:1
Color of the upper-leaf surface	LCA	1, light green; 2, green; 3, dark green
Color of the underside-leaf surface	LCB	1, yellow; 2, greenish yellow; 3, green
Gland color	LGC	1, red; 2, purple or black
Amount of gland on leaf *	LAG	1, one; 2, three to five; 3, more than five
Visible Glands on the upper-leaf surface	LGV	1, non-visible; 2, visible
Leaf venation of the upper-leaf surface	LVU	1, Unapparent; 2, Apparent
Vein began to diverge *	LVD	1, less than 1/3 from leaf base; 2, 1/3 to less than 2/3 from leaf base; 3, 2/3 and more from leaf base
Leaf length	LL	Measured in cm
Leaf width	LW	Measured in cm
Leaf area	LA	Measured in cm^2^
Petiole length	PL	Measured in cm
Amount of golden spots on leaf surface	GS	Number of golden spots
Fruit shape	FSH	1, globose; 2, subglobose; 3, bell shape; 4, ovoid
Maximum width of fruit	FMW	1, less than 1/3 from leaf base; 2, 1/3 to less than 2/3 from leaf base; 3, 2/3 and more from leaf base
Fruit apex shape	FAS	1, acute; 2, round; 3, truncate; 4, cordate
Ratio of fruit length diameter	FDR	1, 1:1; 2, 1.5:1; 3, 2:1
Color of ripening fruit	FCR	1, green to orange; 2, green to pink or red
Scale color	FSC	1, orange; 2, red/brown
Fruit length	FL	Measured in cm
Fruit diameter *	FD	Measured in cm
Fruit stalk length	FS	Measured in cm

* The most important characteristics leading the varietal identification according to Corner [21].

#### 3.4.1. DNA Extraction, Polymerase Chain Reaction (PCR) Protocols

The CTAB-based extraction method was used to extract the genomic DNA of *F. deltoidea* fresh leaves [56]. The absorbance ratio at 260/280 nm measured by using a NanoDrop Lite Microliter Spectrophotometer (Thermo Scientific, Waltham, MA, USA), showed a high purity for the DNA samples ranged between 1.8 and 2. The PCRs were carried out in a total volume of 25 μL for each reaction. The mixture contained 30 ng of DNA, 1 unit Dream Taq DNA polymerase (Thermo Scientific), 10× Dream Taq Green buffer, 2.0 mM, MgCl_2_, 0.2 mM of PCR nucleotide mix (dNTP), and 0.4 μM of primer stock.

DNA amplification was performed using a Thermal Cycler model Techne TC 5000 (Bibby Scientific, Staffordshire, UK). The initial denaturation was 5 min at 94 °C, followed by 35 cycles of 1 min at 94 °C for denaturation, 30 s at 30–51 °C for annealing (according to the primers’ melting temperatures), 1 min at 72 °C for extension, and a final extension cycle of 10 min at 72 °C.

#### 3.4.2. Electrophoresis and Gel Visualization

Electrophoresis separated the PCR amplicons in 1.5% (*w*/*v*) agarose gel. The gels were stained with ethidium bromide, and then visualized under UV light using a Gel Documenter (Carestream, New York, NY, USA). The PCR reactions were repeated twice to test the reproducibility of each primer.

### 3.5. Experimental Design and Statistical Analysis

This experiment was designed based on a Completely Randomized Design (CRD) with ten replicates. Data analyses were implemented on the basis of the molecular and morphological characteristics separately, using different statistical software. The SPSS software version 22 was employed to perform the statistical analyses such as one-way analysis of variance (ANOVA). The mean comparison of the quantitative data and their standard deviation in each accession were calculated using the Least Significant Difference (LSD) method at *p* ≤ 0.01.

#### 3.5.1. PCA and Cluster Analysis Based on the Morphological Data

Prior to cluster analysis, the means of quantitative characters were standardized to eliminate the effects of different scales of measurement. In order to visualize the phylogenetic relationships between the accessions, cluster analysis was conducted on the basis of the Unweighted Pair Group Method with Arithmetic Mean (UPGMA) clustering method. The NTSYSpc software version 2.1 [57] was used to estimate the Euclidean distance matrices and the Jaccard’s similarity coefficients as well. The JMP 8 software (SAS Institute Inc., 2009) was utilized to perform the cluster analyses, to generate the related dendrograms and color maps, as well as the Eigenvalues [58]. Principal Components Analysis (PCA) and cumulative percentage of the principal components (PCs) was confirmed twice, using the JMP 8 and SPSS software. The scree plots and biplot were drawn using the Minitab 17 and MVSP version 3.1 (Kovach Computing Services, Isle of Anglesey, UK) statistical software, respectively.

#### 3.5.2. Cluster Analysis Based on the Molecular Data

Estimation of the Jaccard’s similarity coefficients [39] based on the molecular characteristics (binary data) was done by NTSYSpc version 2.1 [57]. The dendrograms and color maps of the cluster analysis were generated by the JMP 8 software for the molecular data as well. The Shannon indices were estimated by using the POPGENE 32 software.

#### 3.5.3. STRUCTURE Analysis

Genetic clustering algorithms require a certain amount of data to produce informative results [59]. To this end, prior to running the structure analysis, the 30 accessions were grouped into nine distinct populations. Each group was given a code from 1 to 9, so that the individuals assigned to a particular variety were allowed to be considered as a distinct population by varying their location, as described by Hubisz *et al.* [59]. To examine the genetic composition of the individuals based on their ISSR genotypes, the Bayesian clustering software STRUCTURE version 2.3.4 [60,61] was used to assign the individuals to a given number of (*K*) populations. The most likely number of populations (*K*) was estimated under the admixture model and correlated allele frequencies, with no prior information on population origin [62]. The program was run with a burn-in period of 100,000 iterations followed by 50,000 Markov Chain Monte Carlo (MCMC) iterations. Ten independent runs were performed under the admixture model for *K* = 1–9 clusters. The results of the 10 independent runs were averaged for each *K* value to determine the most likely model, *i.e.*, the one with the highest likelihood [44]. For this purpose, the produced files of the structure analysis were compressed into a single “rar” file and were then submitted to the web-based software Structure Harvester 0.6.93 version [63], to identify the average log likelihood, Ln P(D), and the best K following the Δ*K*-method [45].

## 4. Conclusions

ISSR markers are recently used with an emphasis on genetic diversity and population structure [64]. The results of the present investigation proved the efficacy of morphological and molecular markers in studying the genetic variation of the *Ficus deltoidea* population. This study also was convincing on the reliability of the qualitative characteristics in discriminating *F. deltoidea* varieties using PCA and cluster analyses compared to the quantitative and molecular (ISSR) data. Nevertheless, the ISSRs were found more efficient in revealing the evolutionary events, as well as the genetic structure of the *F. deltoidea* population in Peninsular Malaysia. The competence of the methods used in the current study could be observed in the results produced. However, the use of a higher number of ISSRs in combination with codominant markers such as SSRs is strongly recommended. In fact, environmental factors affect phenotypic plasticity of any species more than their DNA composition. Therefore, the low intraspecific genetic diversity of *F. deltoidea* population in Malaysia revealed by ISSR markers should be taken into consideration. Furthermore, the biodiversity and conservation of this valuable species should be seriously considered in the future.

## References

[1] Kamarudin K.R., Rehan A.M., Hashim R., Usup G. (2010). An update on diversity of sea cucumbers (*Echinodermata: Holothuroidea*) in Malaysia. Malays. Nat. J..

[2] Hezri A.A., Hasan M.N. (2006). Towards sustainable development? The evolution of environmental policy in Malaysia. Nat. Resour. Forum.

[3] Kochummen K.M., Rusea G. (2000). Moraceae. Tree Flora Sabah Sarawak.

[4] Bunawan H., Amin N.M., Bunawan S.N., Baharum S.N., Noor N.M. (2014). *Ficus Deltoidea* Jack: A review on its phytochemical and pharmacological importance. Evid. Based Complement. Altern. Med..

[5] Draman S., Aris M.A., Akter S.F.U., Azlina H., Nor A., Muzaffar R., Norazlanshah H. (2012). Mas Cotek (*Ficus deltoidea*): A Possible supplement for type II diabetes: A pilot study. Pertanika J. Trop. Agric. Sci..

[6] Adam Z., Hamid M., Ismail A., Khamis S. (2007). Effect of *Ficus deltoidea* aqueous extract on blood glucose level in normal and mild diabetic rats. Malays. J. Health Sci..

[7] Aminudin N., Sin C.Y., Chee E.S., Nee K.I., Renxin L. (2007). Blood glucose lowering effect of *Ficus deltoidea* aqueous extract. Malays. J. Sci..

[8] Hakiman M., Maziah M. (2009). Non-enzymatic and enzymatic antioxidant activities in aqueous extract of *Ficus deltoidea* accessions. J. Med. Plant Res..

[9] Misbah H., Azlina A.A., Aminudin N. (2013). Antidiabetic and antioxidant properties of *Ficus deltoidea* fruit extracts and fractions. BMC Complement. Altern. Med..

[10] Sulaiman M.R., Hussain M.K., Zakaria Z.A., Somchit M.N., Moin S., Mohamad A.S., Israf D.A. (2008). Evaluation of the antinociceptive activity of *Ficus deltoidea* aqueous extract. Fitoterapia.

[11] Abdulla M.A., Ahmed K.A.A., Abu-Luhoom F.M., Muhanid M. (2010). Role of *Ficus deltoidea* extract in the enhancement of wound healing in experimental rats. Biomed. Res..

[12] Berg C.C. (2003). Flora Malesiana precursor for the treatment of Moraceae 3: Ficus subgenus Ficus. Blumea.

[13] Fatihah H.N.N., Mat N., Zaimah A.R., Zuhailah M.N., Norhaslinda H. (2012). Morphological phylogenetic analysis of seven varieties of *Ficus deltoidea* Jack from the Malay Peninsula of Malaysia. PLoS ONE.

[14] Berg C.C., Corner E.J. (2005). Moraceae (*Ficus*). Ser. I Seed Plants Flora Malays..

[15] Mat N., Rosni N.A., Ab Rashid N.Z., Haron N., Nor Z.M., Nudin N.F.H., Yunus A.G., Ali A.M. (2012). Leaf morphological variations and heterophylly in *Ficus deltoidea* Jack (Moraceae). Sains Malays..

[16] Valdiani A., Kadir M.A., Saad M.S., Talei D., Tan S.G. (2012). Intra-specific hybridization: Generator of genetic diversification and heterosis in *Andrographis paniculata* Nees. A bridge from extinction to survival. Gene.

[17] Dehghan-Shoar M., Hampton J.G., Hill M.J. (2005). Identifying and discriminating among Lucerne cultivars using plant morphological characters. N. Z. J. Agric. Res..

[18] Bhore S.J., Nurul A.H., Shah F.H. (2009). Genetic variability based on randomly amplified polymorphic DNA in mistletoe fig (*Ficus deltoidea* Jack) collected from Peninsular Malaysia. J. For. Sci..

[19] Foll M., Gaggiotti O. (2006). Identifying the environmental factors that determine the genetic structure of populations. Genetics.

[20] Valdiani A., Talei D., Javanmard A., Tan S.G., Kadir M.A., Maziah M. (2014). Morpho-molecular analysis as a prognostic model for repulsive feedback of “*Andrographis paniculata*” to allogamy. Gene.

[21] Corner E. (1969). The complexity of *Ficus deltoidea*; A recent invasion of the Sunda Shelf. Philos. Trans. R. Soc..

[22] Went F.W. (1953). The effect of temperature on plant growth. Annu. Rev. Plant Physiol..

[23] Morison J.I.L., Morecroft M.D. (2006). Plant Growth and Climate Change.

[24] Talei D., Valdiani A., Khanif Y.M., Abdullah M.P. (2013). Estimation of salt tolerance in *Andrographis paniculata* accessions using multivariate regression model. Euphytica.

[25] Iezzoni A.F., Pritts M.P. (1991). Applications of principal components analysis to horticultural research. Hortic. Sci..

[26] Mars M., Marrakchi M. (1999). Diversity of pomegranate (*Punica granatum* L.) germplasm in Tunisia. Genet. Resour. Crop Evol..

[27] Upadhyaya H.D., Gowda C.L., Buhariwalla H.K., Crouch J.H. (2006). Efficient use of crop germplasm resources: Identifying useful germplasm for crop improvement through core and mini-core collections and molecular marker approaches. Plant Genet. Resour. Character Util..

[28] Goodman M.M. (1972). Distance analysis in biology. Syst. Zool..

[29] Schut J.W., Qi X., Stam P. (1997). Association between relationship measures based on AFLP markers, pedigree data and morphological traits in barley. Theor. Appl. Genet..

[30] Papadopoulou K., Ehaliotis C., Tourna M., Kastanis P., Karydis I., Zervakis G. (2002). Genetic relatedness among dioecious *Ficus carica* L. cultivars by random amplified polymorphic DNA analysis, and evaluation of agronomic and morphological characters. Genetica.

[31] Saddoud O., Baraket G., Chatti K., Trifi M., Marrakchi M., Salhi-Hannachi A., Mars M. (2008). Morphological variability of Fig (*Ficus carica* L.) cultivars. Int. J. Fruit Sci..

[32] Podgornik M., Vuka I., Vrhovnik I., Mavsar D. (2010). A survey and morphological evaluation of fig (*Ficus carica* L.) genetic resources from Slovenia. Sci. Hortic..

[33] Hardig T.M., Brunsfeld S.J., Fritz R.S., Morgan M., Orians C.M. (2000). Morphological and molecular evidence for hybridization and introgression in a willow (Salix) hybrid zone. Mol. Ecol..

[34] Ackermann R.R., Bishop J.M. (2010). Morphological and molecular evidence reveals recent hybridization between gorilla taxa. Evolution.

[35] Ruas P.M., Ruas C.F., Rampim L., Carvalho V.P., Ruas E.A., Sera T. (2003). Genetic relationship in *Coffea* species and parentage determination of interspecific hybrids using ISSR (Inter-Simple Sequence Repeat) markers. Genet. Mol. Biol..

[36] Harper F.M., Hart M.W. (2007). Morphological and phylogenetic evidence for hybridization and introgression in a sea star secondary contact zone. Invertebr. Biol..

[37] Neophytou C. (2014). Bayesian clustering analyses for genetic assignment and study of hybridization in oaks: Effects of asymmetric phylogenies and asymmetric sampling scheme. Tree Genet. Genomes.

[38] Liu J., Shi S., Chang E., Yang W., Jiang Z. (2013). Genetic diversity of the critically endangered thuja sutchuenensis revealed by ISSR markers and the implications for conservation. Int. J. Mol. Sci..

[39] Jaccard P. (1901). A comparative study of the floral distribution in Alps and Jura. Bull. Walden Soc. Nat. Sci..

[40] Tairo F., Mneney E., Kullaya A. (2008). Morphological and agronomical characterization of sweet potato (*Ipomoea batatas* (L.) Lam.) germplasm collection from Tanzania. Afr. J. Plant Sci..

[41] Sokal R.R., Micheners C.D. (1958). A statistical method for evaluating systematic relationships. Univ. Kans. Sci. Bull..

[42] Dalirsefat S.B., da Silva Meyer A., Mirhoseini S.Z. (2009). Comparison of similarity coefficients used for cluster analysis with amplified fragment length polymorphism markers in the silkworm, *Bombyx mori*. J. Insect Sci..

[43] Mantel N. (1967). The detection of disease clustering and generalized regression approach. Cancer Res..

[44] Gao X.Y., Zhi X.Y., Li H.W., Klenk H.P., Li W.J. (2014). Comparative genomics of the bacterial genus streptococcus illuminates evolutionary implications of species groups. PLoS ONE.

[45] Earl D.A., von Holdt B.M. (2012). STRUCTURE HARVESTER: A website and program for visualizing STRUCTURE output and implementing the Evanno method. Conserv. Genet. Resour..

[46] Egger B., Sefc K.M., Makasa L., Sturmbauer C., Salzburger W. (2012). Introgressive hybridization between color morphs in a population of cichlid fishes twelve years after human-induced secondary admixis. J. Hered..

[47] Ramirez B.W. (1970). Host specificity of fig wasps (Agaonidae). Evolution.

[48] Yu H., Compton S.G. (2012). Moving your sons to safety: Galls containing male fig wasps expand into the centre of figs, away from enemies. PLoS ONE.

[49] Starr F., Starr K., Loope L. (2003). Ficus deltoidea, Mistletoe Fig (Moraceae).

[50] Harrison R.D. Figs and Fig Wasps: An Intricate Interaction. Proceeding of the International Field Biology Course.

[51] McKey D. (1989). Population biology of figs: Applieations for conservation. Experientia.

[52] Poore M.E.D. (1986). Studies in Malaysian rain forest I. The forest on triassic sediments in Jengka Jorest Reserve. J. Ecol..

[53] Ranc N., Muños S., Santoni S., Causse M. (2008). A clarified position for *Solanum lycopersicum* var. *cerasiforme* in the evolutionary history of tomatoes (solanaceae). BMC Plant Biol..

[54] IPGRI, CIHEAM (2003). Descriptors for Fig; International Plant Genetic Resources Institute, Rome Italy, and International Centre for Advanced Mediterranean Agronomic Studies.

[55] Pandey S.K., Singh H. (2011). A simple, cost-effective method for leaf area estimation. J. Bot..

[56] Doyle J.J., Doyle J.L. (1990). Isolation of plant DNA from fresh tissue. Focus.

[57] Rohlf F.J. (2000). NTSYSpc: Numerical Taxonomy System. Ver. 2.1. Applied Biostatic, Exeter Software.

[58] SAS Institute Inc. (2009). JMP^®^ 8 User Guide.

[59] Hubisz M.J., Falush D., Stephens M., Pritchard J.K. (2009). Inferring weak population structure with the assistance of sample group information. Mol. Ecol. Resour..

[60] Pritchard J.K., Stephens M., Donnelly P. (2000). Inference of population structure using multilocus genotype data. Genetics.

[61] Falush D., Stephens M., Pritchard J.K. (2007). Inference of population structure using multilocus genotype data: Dominant markers and null alleles. Mol. Ecol. Notes.

[62] Tacuatiá L.O., Eggers L., Kaltchuk-Santos E., Souza-Chies T.T. (2012). Population genetic structure of *Sisyrinchium micranthum* Cav. (Iridaceae) in Itapuã State Park, Southern Brazil. Genet. Mol. Biol..

[63] Evanno G., Regnaut S., Goudet J. (2005). Detecting the number of clusters of individuals using the software STRUCTURE: A simulation study. Mol. Ecol..

[64] Zhang F., Ge Y., Wang W., Yu X., Shen X., Liu J., Liu X., Tian D., Shen F., Yu Y. (2012). Molecular characterization of cultivated bromeliad accessions with inter-simple sequence repeat (ISSR) markers. Int. J. Mol. Sci..

